# Innate Immunity Induced by the Major Allergen Alt a 1 From the Fungus *Alternaria* Is Dependent Upon Toll-Like Receptors 2/4 in Human Lung Epithelial Cells

**DOI:** 10.3389/fimmu.2018.01507

**Published:** 2018-07-30

**Authors:** Tristan Hayes, Amanda Rumore, Brad Howard, Xin He, Mengyao Luo, Sabina Wuenschmann, Martin Chapman, Shiv Kale, Liwu Li, Hirohito Kita, Christopher B. Lawrence

**Affiliations:** ^1^Department of Biological Sciences, Virginia Tech, Blacksburg, VA, United States; ^2^Department of Pediatrics, School of Medicine, Indiana University Bloomington, Indianapolis, IN, United States; ^3^Department of Biology, Randolph College, Lynchburg, VA, United States; ^4^Indoor Biotechnologies, Charlottesville, VA, United States; ^5^Biocomplexity Institute, Virginia Tech, Blacksburg, VA, United States; ^6^Division of Allergic Diseases, Internal Medicine, Mayo Clinic, Rochester, MN, United States

**Keywords:** *Alternaria*, allergen, innate immunity, toll-like receptors, mold, fungus–host interaction

## Abstract

Allergens are molecules that elicit a hypersensitive inflammatory response in sensitized individuals and are derived from a variety of sources. Alt a 1 is the most clinically important secreted allergen of the ubiquitous fungus, *Alternaria*. It has been shown to be a major allergen causing IgE-mediated allergic response in the vast majority of *Alternaria*-sensitized individuals. However, no studies have been conducted in regards to the innate immune eliciting activities of this clinically relevant protein. In this study, recombinant Alt a 1 was produced, purified, labeled, and incubated with BEAS-2B, NHBE, and DHBE human lung epithelial cells. Alt a 1 elicited strong induction of IL-8, MCP-1, and Gro-a/b/g. Using gene-specific siRNAs, blocking antibodies, and chemical inhibitors such as LPS-RS, it was determined that Alt a 1-induced immune responses were dependent upon toll-like receptors (TLRs) 2 and 4, and the adaptor proteins MYD88 and TIRAP. Studies utilizing human embryonic kidney cells engineered to express single receptors on the cell surface such as TLRs, further confirmed that Alt a 1-induced innate immunity is dependent upon TLR4 and to a lesser extent TLR2.

## Introduction

Besides being a common cause of allergic rhinitis, sensitivity to the airborne fungus *Alternaria alternata* is believed to be a common cause of allergic/atopic asthma. Epidemiological studies from locations worldwide indicate that *Alternaria* sensitivity is closely linked with the development of asthma and up to 70% of mold-allergic patients have skin prick test (SPT) reactivity to *Alternaria* (1–3). *Alternaria* sensitivity has been shown to not only be a risk factor for asthma but can also directly lead to the development of severe and potentially fatal asthma often more than any other fungus (1–5). In addition, *Alternaria* sensitization has been determined to be one of the most important factors in the onset of childhood asthma in the southwest desert regions of the US and other arid regions in the world (2, 6, 7). *Alternaria* spores are ubiquitous, routinely found in atmospheric surveys in the US and in other countries and are the most frequently encountered fungal spore type (8). Airborne spore counts are often 1,000-fold greater than pollen counts, and exposures are often longer in duration. Indeed, it has long been speculated that this type of exposure may be partially responsible for both the chronic nature and severity of asthma in *Alternaria-*sensitized individuals (9). Indeed, in a survey by the National Institute of Environmental Health & Safety of 831 homes, containing 2,456 individuals, it was found that the prevalence of current symptomatic asthma correlated with increasing indoor *Alternaria* concentrations (3). Higher levels of *Alternaria* antigens in the environment significantly increased odds of having had asthma symptoms during the preceding year, more so than other examined antigens.

Although some research has been performed on the physiological and molecular identification of *Alternaria* allergens, approximately three major and five minor allergenic proteins have been described to date (10, 11). In general, the biological role of these allergens and other fungal products in the development of allergy and asthma is poorly understood. There is clearly a need to elucidate the role of *Alternaria* immunoreactive proteins and other molecules in the development of asthma from mechanistic perspectives.

Many of the known *Alternaria* allergens are intracellular proteins with clinically relevant homologs being reported in other fungi with known functions such as enolase, ribosomal proteins, nuclear transport factor, and aldehyde dehydrogenase to name a few (11–13). Alt a 1, the major allergen produced by *Alternaria* spp. namely *A. alternata*, is a relatively small (157 amino acids) secreted protein with no clear function in fungal metabolism or ecology (14, 15). Its protein sequence and β-barrel structure is unique among fungal allergens with no known cross reactivity to other allergens (16–20). Diagnosis of *A. alternata* sensitization is often hampered by the variability and complexity of fungal extracts, and thus simplification of the diagnostic procedures with purified allergens has been investigated. Currently, in some allergy clinics in the US, pure Alt a 1 protein is often used to assess sensitization in SPTs in lieu of total fungal extract because it produces the same reaction as total antigen extracts in the majority (80–90%) of human subjects (21–23). Furthermore, Alt a 1, either in its natural or recombinant form, is sufficient for a reliable diagnosis of *A. alternata* sensitization and induces skin prick reactivity comparable with that produced by commercially available *A. alternata* extract (21–23).

In this study, we investigated and report for the first time the innate immunostimulatory activities of Alt a 1 in human bronchial epithelial cells. We found Alt a 1 has potent cytokine and chemokine inducing activity. Moreover, this activity was found to be dependent upon toll-like receptors (TLR2 and TLR4) and associated signaling pathways. This study is the very first in regards to defining the potential role of a single purified *Alternaria* product or protein in innate immunity. Results of these studies are discussed.

## Materials and Methods

### Vector Construction and Transformation of *Pichia pastoris*

An Alt a 1 cDNA harboring vector (pGAPZ, Thermo Fisher Scientific, Waltham, MA, USA) for expression in *P. pastoris* was provided as a generous gift from Dr. Martin Chapman (Indoor Biotechnologies, Charlottesville, VA, USA). Briefly, the pGAPZ-Alt a 1 vector contained a 6× poly-histidine tag for purification *via* immobilized metal ion affinity chromatography (IMAC) and allowed for zeocin to be used for selection. The pGAPZ-Alt a 1 plasmid was transformed into *P. pastoris* GS115 (Thermo Fisher Scientific, Waltham, MA, USA) *via* heat shock and plated on media containing zeocin according to the manufacturer’s protocols (Thermo Fisher Scientific, Waltham, MA, USA). Next, as per the manufacturer’s protocols, zeocin-resistant *P. pastoris* colonies were then screened for the presence of Alt a 1 using colony-based PCR using forward primer 5′-gtctggaagatctccgagttttacggacgcaag-3′ and the reverse primer 5′-cttgcgtccgtaaaactcggagatcttccagac-3′. Positive colonies were selected and used for downstream expression and production of rAlt a 1 in *P. pastoris* GS115.

### Protein Expression and Purification

The rAlt a 1 protein was expressed in *P. pastoris* GS115 according to the manufacturer’s instructions (Thermo Fisher Scientific, Waltham, MA, USA) and purification followed a typical IMAC protocol (Qiagen Inc., Valencia, CA, USA). Briefly, yeast was grown in 500 mL yeast extract peptone dextrose broth at 22°C while shaking at 180 RPM. After 60 h, the culture media was separated into cells and supernatant by centrifuge at 5,000 × *g* for 10 min. The supernatant was then buffer exchanged with 2 L of lysis buffer (50 mM NaH_2_PO_4_, 500 mM NaCl, 30 mM Imidazole, pH 8.0). After concentrating to 25 mL, supernatant was then applied to NiNTA resin (Qiagen Inc., Valencia, CA, USA) that had been washed and equilibrated in lysis buffer per manufacturer’s protocols. Four column volumes of lysis buffer were flowed through the column. Next, 5 mL elution buffer (50 mM NaH_2_PO_4_, 500 mM NaCl, 50 mM imidazole, pH 8.0) was applied to the column. Elution buffer with increasing imidazole concentrations (100, 150, and 200 mM, respectively) was then applied. rAlt a 1 protein eluted at 200 mM imidazole concentration.

Purity was assessed *via* 15% SDS-polyacrylamide gel electrophoresis. As expected, the rAlt a 1 protein appeared to be a heterodimer consisting of 14.4 and 17 kDa bands under denaturing conditions. Amicon Ultra Centrifugal Filters MWCO 10 kDA (Sigma-Aldrich, St. Louis, MO, USA) were used to concentrate proteins, and proteins were buffer exchanged with endotoxin-free PBS, pH 7.4 (Thermo Fisher Scientific, Waltham, MA, USA) for downstream endotoxin removal and applications. Approximately 20 mg/L of rAlt a 1 was typically obtained following purification.

### Endotoxin Removal and Quantification of rAlt a 1

Even though protein was produced in yeast, potential endotoxin contamination was removed from purified Alt a 1 using endotoxin removal columns (Detoxi-Gel endotoxin removing columns, Thermo Fisher Scientific, Waltham, MA, USA). Briefly, resin was equilibrated in 1% sodium deoxycholate followed by five volume washes of PBS. 1 mL of protein was loaded onto the column and incubated for 1 h. Protein was eluted by addition of endotoxin-free PBS, pH7.4 (Thermo Fisher Scientific, Waltham, MA, USA).

Quantification of endotoxin levels was performed using an enzyme-linked immunosorbent assay (ELISA) Kit (Biomatik, ON, Canada). Briefly, 50 µL of protein was assessed following the manufacturer’s protocol. Samples were run in duplicate in 96-well plate format and were read on a Versa MAX ELISA Microplate Reader (Molecular Devices, Sunnyvale, CA, USA) at room temperature. Measurements at OD_450_ were corrected against values obtained at OD_570_ following the manufacturer’s suggestions. A standard curve was generated for each reading and generated using R software. Endotoxin/LPS concentration was determined using the standard curve and tabulated in ng/mL of LPS. Experiments were performed at least five times/protein preparation. In all experiments, purified rAlt a 1 contained below the detectable limit of endotoxin (<0.01 ng/mL). After testing for homogeneity of variances, Tukey’s HSD was performed and adjusted (if applicable).

### Membrane-Based Cytokine Arrays

Human cell culture supernatants were assayed for general secretion of different cytokines and chemokines using the RayBio C-Series Human Cytokine Antibody Array C1 per manufacturer’s protocols (RayBiotech, Norcross, GA, USA). BEAS-2B cells were grown in DMEM + 1% Pen/Strep/10% FBS. NHBE/DHBE were grown in BEGM media per the manufacturer’s protocols (Lonza, Walkersville, MD, USA). Cells were incubated at 37°C/5% CO_2_. Approximately 4 × 10^6^ BEAS-2B (ATCC CRL 9609, Manassas, VA, USA) or 4 × 10^6^ NHBE/DHBE (Lonza, Walkersville, MD, USA) cells were starved for 4 h prior to the addition of 50 µg rAlt a 1 and incubated for 24 h. Supernatants were collected and used in downstream experiments per the manufacturer’s protocols (RayBiotech, Norcross, GA, USA). Briefly, membranes were blocked with the blocking buffer and then washed. The membranes were then treated with the samples for 2 h at RT shaking at 90 RPM. After an additional wash, the biotinylated antibody cocktail was used to cover the membranes. After a 2-h incubation at RT with shaking at 90 RPM another wash step was conducted. Afterward, the membranes were covered with horseradish peroxidase streptavidin concentrate and incubated for 2 h at RT with shaking at 90 RPM. The membranes were then washed and signals were detected using chemiluminescence. Briefly, membranes were washed with two detection buffers provided by RayBiotech (Norcross, GA, USA) and exposed continuously from 5 to 600 s with images taken at multiple intervals in between using BioRad Chemi Doc CRS+ System with Image Lab Software (BioRad, Berkeley, CA, USA). Images were then exported with no correction or image modification.

### Enzyme-Linked Immunosorbent Assays

Enzyme-linked immunosorbent assays were performed using Human IL-8, MCP-1, and GRO (a/b/g) ELISA MAX (BioLegend, San Diego, CA, USA) kits. Supernatants from cells treated with rAlt a 1 were examined following the manufacturer’s protocol. Samples were run in duplicate in 96-well plate format and were read on a Versa MAX ELISA Microplate Reader (Molecular Devices, Sunnyvale, CA, USA) at room temperature. Measurements at OD_450_ were corrected against values obtained at OD_570_ following the manufacturer’s suggestions. A standard curve was generated for each reading and generated using R software. Cytokine/chemokine concentrations were determined using the standard curve and tabulated in pg/mL. Experiments were performed at least five times. After testing for homogeneity of variances, Tukey’s HSD was performed and adjusted (if applicable).

### Human Embryonic Kidney (HEK) 293 Cells Engineered to Express TLRs and Measurement of NF-κB Activity

Human embryonic kidney 293-Blue hTLR4 cells expressing TLR4, MD2/CD14 co-receptor genes and a secreted embryonic alkaline phosphatase (SEAP) reporter gene under control of an IL-12 promoter and HEK-Blue Null 2 cells lacking TLR4 receptor but expressing the IL-12 promoter and SEAP reporter gene (Invivogen, San Diego, CA, USA) were seeded in a 96-well plate. The final volume of culture media was 200 µL DMEM + 1% Pen/Strep/10% FBS. After 16 h at 37°C/5% CO_2_, the media was removed from the cells. Cells were then starved for 2 h in 200 µL DMEM + 1% Pen/Strep. Cells were pretreated with 5 ng/mL ultrapure LPS-RS (Invivogen, San Diego, CA, USA), 10 µg/mL anti-hTLR4-IgG or 10 µg/mL mouse IgG 1 control antibody (antibodies from Invivogen, San Diego, CA, USA) for 1 h. Then cells were treated with 1 µg rAlt a 1. Cells were then incubated at 37°C/5% CO_2_ for 24 h. Afterward 20 µL of the cell supernatant was added to 180 µL QUANTI-Blue reagent (Invivogen, San Diego, CA, USA). After incubation for 3 h at 37°C, the plate was read at 655 nM (VersaMax ELISA microplate reader) at RT. After testing for homogeneity of variances, Tukey’s HSD was performed and adjusted (if applicable).

Human embryonic kidney 293-Blue hTLR2 cells expressing TLR2 and CD14 co-receptor genes and an SEAP reporter gene under control of the interferon (IFN)_β_ minimal promoter were seeded in a 96-well plate, final volume was 200 µL DMEM + 1% Pen/Strep/10% FBS. After 16 h at 37°C/5% CO_2_, the media was removed from the cells. Cells were then starved for 2 h in 200 µL DMEM + 1% Pen/Strep. Cells were pretreated with 10 μg/mL human IgA2-control, 10 µg/mL anti-hCD14-IgA, and 1 μg/mL Mab-hMD2 (all from Invivogen, San Diego, CA, USA), for 2 h. Then cells were treated with 1 µg rAlt a 1. Cells were then incubated at 37°C/5% CO_2_, for 24 h. Afterward 20 µL of the cell supernatant was added to 180 µL QUANTI-Blue reagent (Invivogen, San Diego, CA, USA). After incubation for 3 h at 37°C, the plate was read at 655 nM (VersaMax ELISA microplate reader) at RT. After testing for homogeneity of variances, Tukey’s HSD was performed and adjusted (if applicable).

Human embryonic kidney 293-Blue hTLR5 cells expressing TLR5 gene and an SEAP reporter gene under control of an AP-1 promoter were seeded on a 96-well plate. Cells were treated with 100 ng/mL of FLA-ST Ultrapure (Invivogen, San Diego, CA, USA) or 1 µg rAlt a 1. This purified flagellin from *Salmonella typhimurium* is detected by TLR5 resulting in MyD88-mediated NF-κB activation. Cells were incubated overnight at 37°C/5% CO_2_. After incubation, 20 µL of the cell supernatant was added to 180 µL QUANTI-Blue reagent (Invivogen). After incubation for 3 h at 37°C, the plate was read at 655 nM (VersaMax ELISA microplate reader) at RT. After testing for homogeneity of variances, Tukey’s HSD was performed and adjusted (if applicable).

### Blocking Antibodies in BEAS-2B Cells

2.5 × 10^5^ BEAS-2B cells were plated in 1 mL DMEM + 1% Pen/Strep/10% FBS. After 24 h, 10 µg/mL of either anti-hTLR2-IgA, 10 µg/mL anti-hTLR4-IgG, 10 µg/mL human IgA2-control, or 10 µg/mL mouse-IgG1-control (all from Invivogen, San Diego, CA, USA) was added. After 2 h, 100 µg rAlt a 1 was added. Cells were then incubated for 24 h at 37°C/5% CO_2_. Supernatants were then collected and assayed for cytokines *via* ELISA as described previously.

### Gene Knockdown Using siRNAs in BEAS-2B Cells

3.0 × 10^5^ BEAS-2B cells were seeded and cultured to 70% confluency in 24-well plates containing DMEM with 10% FBS. The cells were then transfected with 10 nM of either scrambled control, TLR2, TLR4, TIRAP, or MyD88 siRNAs (Santa Cruz Biotechnology, Santa Cruz, CA, USA) in serum-free Opti-MEM medium (Invitrogen, Carlsbad, CA, USA) using Lipofectamine RNA iMAX Reagent (Invitrogen, Carlsbad, CA, USA) according to the manufacturer’s protocols. After 24 h, cells were treated with either 50 µg rAlt a 1 or where appropriate, 5 ng/mL of LPS (Sigma-Aldrich, St. Louis, MO, USA). To determine efficacy of gene silencing, western blot analysis was performed 24 h after transfection.

### Statistical Analysis

Data are expressed as means and SDs. Data were tested for homogeneity of variances and appropriated analysis of variances tests were performed and adjusted accordingly. Downstream analysis of all numerical data utilized R software and packages. Packages used for analyzing data included Coin, Car, Drc, Multcomp, and Sandwich (24–28).

## Results

### Alt a 1 Induces Innate Immune Responses in BEAS-2B Bronchial Epithelial Cells

**Figure 1:**
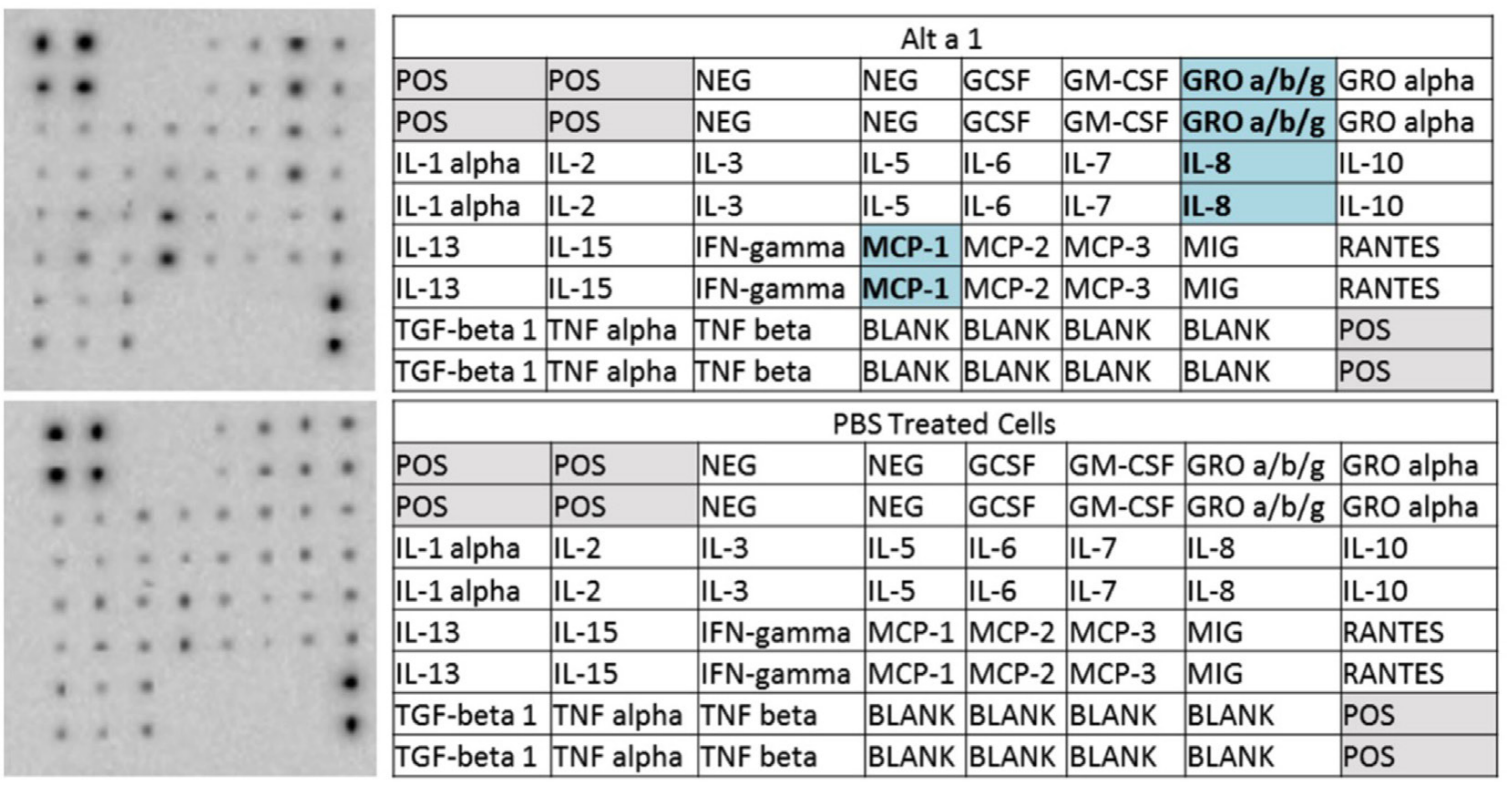
Cytokine profiling of rAlt a 1 treated BEAS-2B cells. 5.0 × 10^5^ BEAS-2B cells were treated with 50 µg rAlt a 1 or 50 µL of endotoxin-free, sterile PBS (negative control). Cytokine array blots (*top left*) and corresponding maps of targets (*top right*) derived from cells treated with rAlt a 1, cytokine array blot (*bottom left*) and corresponding map of targets derived from cells treated with PBS (*bottom right*). Comparisons were made between the two blots and cytokines and chemokines highlighted in blue are those that were found to be qualitatively stronger in cells treated with rAlt a 1.

To initially characterize the innate immune response induced by rAlt a 1 in bronchial epithelial cells *in vitro*, we used human cytokine/chemokine arrays harboring antibodies corresponding to 23 target molecules. Our results indicated that Alt a 1 induced the secretion of several cytokines and chemokines in BEAS-2B human bronchial epithelial airway cells—primarily MCP-1 (CCL2), IL-8, and GRO-a/b/g (CXCL1/2/3) (Figure 1). Similar results were obtained in experiments using NHBE and DHBE cells (data not shown). There were no marked differences between responses in BEAS-2B, NHBE, and DHBE cells. There was some indication that IL-15 may also be inducible by Alt a 1 in our array studies but was not explored further.

**Figure 2:**
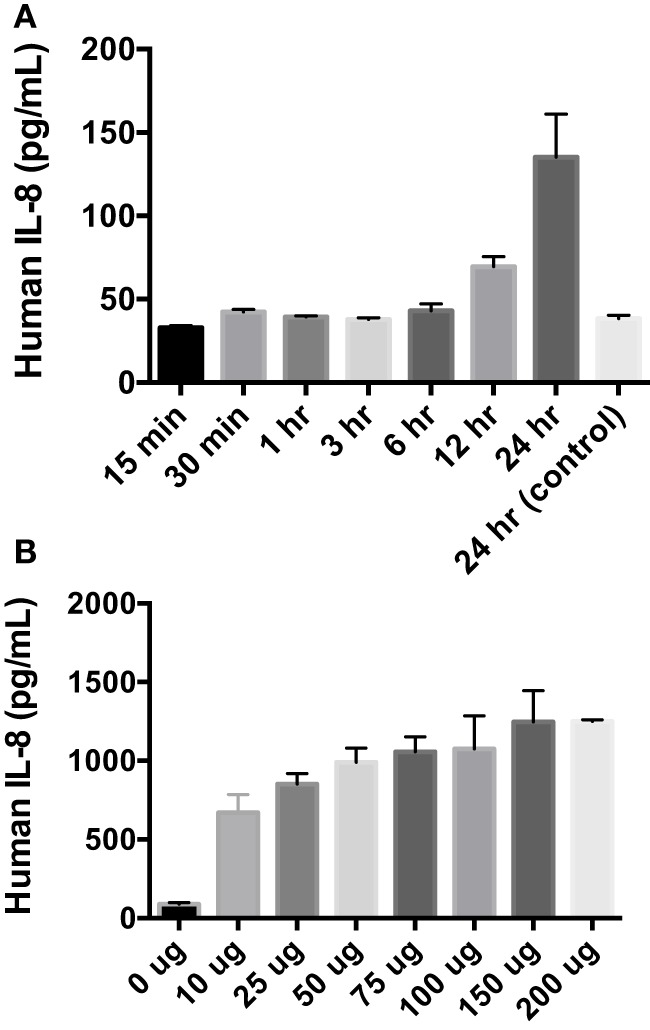
Temporal and dose dependency of Alt a 1 induced IL-8 in BEAS-2B cells. **(A)** 2.0 × 10^5^ BEAS-2B cells were grown in RPMI + FBS + 1% Pen Strep. Cells were starved for 6 h prior to induction with rAlt a 1 or endotoxin-free sterile PBS. The starve media consisted of RPMI + 1% Pen Strep without FBS. Cells were treated with 50 µg rAlt a 1 for the specified time. Supernatants were collected and assayed *via* BIOLEGEND IL-8 ELISAMAX. After testing for homogeneity of variances, Tukey’s HSD was performed and adjusted (*p < 0.001). Data are represented as mean (SD). Comparisons were made between rAlt a 1 treated and untreated cells (PBS-treated 24 h control). **(B)** 5 × 10^5^ BEAS-2B cells were grown in RPMI + FBS + 1% Pen Strep. They were then starved for 6 h. The starve media was RPMI + 1% Pen Strep. Cells were treated with rAlt a 1 or PBS (0 μg) for 24 h. Media was collected and assayed *via* BIOLEGEND IL-8 ELISAMAX. Data are represented as mean (SD). Comparisons were made between Alt a 1-treated and control PBS-treated cells (*p < 0.001).

After finding that rAlt a 1 induces the secretion of several innate immune cytokines and chemokines *via* array blots, determining if there was a time-dependent secretion of IL-8 in BEAS-2B cells was conducted *via* ELISA assay (Figure 2). The goal of this experiment was to characterize the temporal aspects of IL-8 secretion and to determine if 24 h post treatment was optimal for supernatant collection. Human cells were incubated with Alt a 1 for time periods ranging from 15 min to 24 h. Significant increases of IL-8 secretion occurred after cells were incubated with Alt a 1 for 12 h. However, 24 h was the time point at which IL-8 secretion was the highest (Figure 2A). We also determined if Alt a 1 induces IL-8 in a dose-dependent manner and found this to be the case (Figure 2B). We found very strong induction of IL-8 with as little as 10 μg of rAlt a 1 with a maximum induction using 150 μg of protein. This indicates Alt a 1 is quite potent at inducing IL-8 in BEAS-2B cells even at lower concentrations.

**Figure 3:**
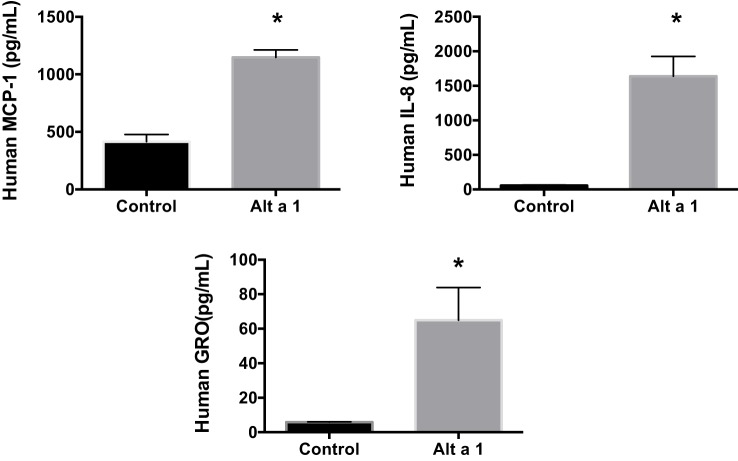
Induction of cytokines and chemokines in BEAS-2B cells treated with Alt a 1. 5.0 × 10^5^ human BEAS-2B cells were treated with 50 µg rAlt a 1 for 24 h. Supernatant was collected and cytokine/chemokine levels were determined using BIOLEGEND ELISAMAX [MCP-1 (CCL2), IL-8, and GRO-a/b/g (CXCL1/2/3)]. Data are represented as mean (SD). After testing for homogeneity of variances, Tukey’s HSD was performed and adjusted (*p < 0.001).

Additional ELISA assays were next used instead to quantify and confirm the levels of secreted cytokines/chemokines, MCP-1 (CCL2), IL-8, and GRO-a/b/g (CXCL1/2/3). Human BEAS-2B cells were treated with rAlt a 1 for 24 h (Figure 3). The ELISA results confirmed the cytokine array blots and showed that levels of human IL-8, MCP-1, and GRO-a/b/g were significantly increased in rAlt a 1 treated cells.

### Gene Knockdown Approaches Using siRNAs Indicate Alt a 1 Induction of Innate Immune Responses Is Dependent Upon TLR Signaling in BEAS-2B Cells

**Figure 4:**
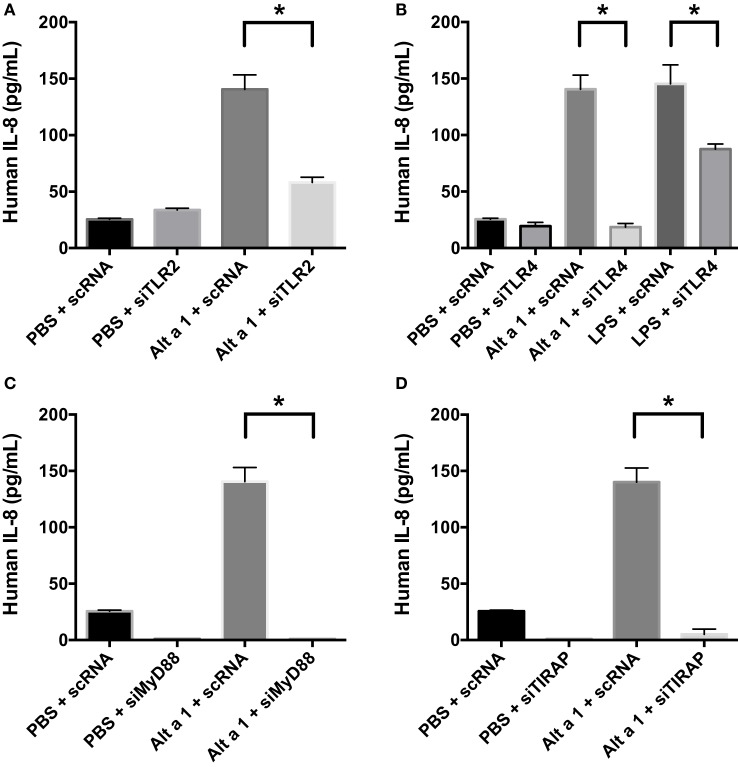
Alt a 1 induces signaling in BEAS-2B cells *via* toll-like receptor (TLR)2, TLR4, MyD88, and TIRAP. 3.0 × 10^4^ human BEAS-2B cells in DMEM + 10% FBS were cultured to 70% confluency in 24-well plates. The cells were transfected with 10 nM of either scrambled control or gene-specific siRNAs in serum-free Opti-MEM medium using Lipofectamine RNA iMAX Reagent according to the manufacturer’s protocols. After 24 h, cells were treated with either 50 µg rAlt a 1 or an equivalent volume of PBS. Media was collected and assayed *via* BIOLEGEND IL-8 ELISAMAX. Data are represented as mean (SD). After testing for homogeneity of variances, Tukey’s HSD was performed and adjusted (if applicable) (*p < 0.001). **(A)** TLR2, **(B)** TLR4, **(C)** MyD88, and **(D)** TIRAP.

To determine the potential role of known pattern recognition receptors (PRRs) such as TLRs and associated signaling pathways in Alt a 1-induced innate immune responses in bronchial epithelial cells (BEAS-2B), a gene knockdown approach using siRNAs was used initially. A suite of gene-specific siRNAs (MyD88, PI-3-K, TIRAP, TLR2, TLR3, TLR4) or scrambled control siRNA (scRNA) were transfected into BEAS-2B cells, then treated with rAlt a 1 for 24 h. We confirmed knockdown of target genes using quantitative reverse transcription PCR. We typically obtained >70% gene-specific knockdown efficiency (data not shown). Supernatants were then collected and assayed for IL-8 levels. These cells showed no consistent reduction in IL-8 secretion when PI-3-K and TLR3 were knocked down (data not shown). In repeated experiments, Alt a 1-treated cells showed decreased IL-8 secretion following incubation with siRNAs corresponding to MyD88, TIRAP, TLR2, and TLR4, compared to scRNA controls (Figure 4). We found that silencing of TLR4 resulted in complete abolishment of Alt a 1 induced IL-8 when compared to scRNA control treated cells. In comparison to silencing of TLR4, the effect of silencing TLR2 was statistically significant but was not as robust in these experiments. Interestingly, akin to our results with TLR4-specific siRNAs, we found that silencing of TIRAP or MyD88 resulted in virtually complete abolishment of Alt a 1 induced IL-8 when compared to scRNA control treated cells indicating the importance of these adaptor proteins downstream of TLR-receptors. Collectively, this data indicated that Alt a 1-induced innate immune responses are dependent upon these receptors and downstream adaptors in BEAS-2B cells (Figure 4).

### Blocking Antibody and Antagonist Approaches Demonstrate Alt a 1 Induction of Innate Immune Responses Is Dependent Upon TLR Signaling in BEAS-2B Cells

We confirmed the results of our siRNA gene knockdown experiments using TLR2 and TLR4 blocking antibodies. In preliminary experiments using TLR4 blocking antibodies, results indicated that TLR4 is important for Alt a 1 induction of MCP-1 (CCL2), IL-8, and GRO-a/b/g (CXCL1/2/3) in BEAS-2B cells (data not shown).

**Figure 5:**
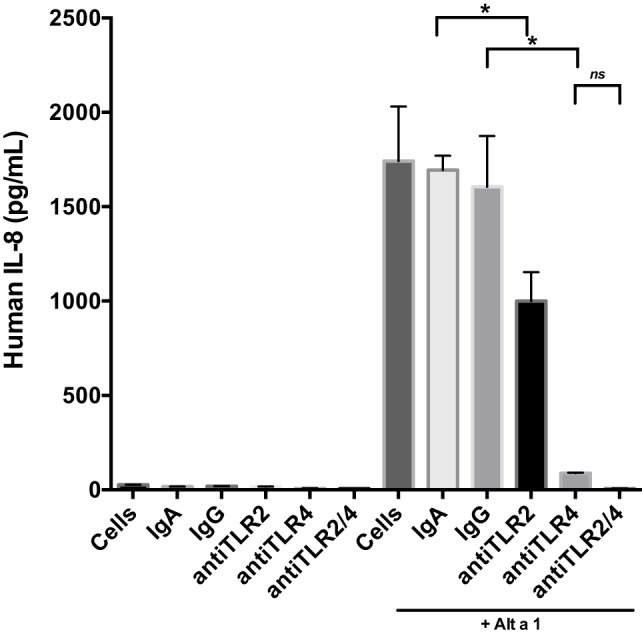
Alt a 1 primarily induces innate immunity through toll-like receptor (TLR)4 in BEAS-2B cells. 2.5 × 10^5^ BEAS-2B cells were grown in RPMI + FPS + 1% Pen Strep. Cells were washed 24 h before use. They were then starved for 6 h. The starve media was RPMI + 1% Pen Strep. Some cells were pretreated with 10 µg/mL anti-hTLR2-IgA, 10 µg/mL anti-hTLR4-IgG, 10 µg/mL human IgA2-control, or 10 µg/mL mouse-IgG1-control for 1 h, then given 50 µg rAlt a 1. Cells incubated for 24 h. Media was collected and assayed *via* BIOLEGEND IL-8 ELISAMAX. Data are represented as mean (SD). After testing for homogeneity of variances, Tukey’s HSD was performed and adjusted (if applicable) (*p < 0.001). The anti-hTLR2-IgA Alt a 1 treatment was compared to the Alt a 1-IgG treated control. The anti-hTLR4-IgG treatment was compared to the Alt a 1-IgG treated control. Alta1 + TLR2/4 treatment is significant when compared to the Alt a 1-IgG treated control or Alta1 + hTLR2 and is designated with an asterisk. However, Alta1 + hTLR2/4 is not significant when compared to Alta1 + TLR4.

Next we incorporated control antibodies (non-blocking), and TLR4 and TLR2 specific blocking antibodies (Figure 5). Blocking TLR4 resulted in a much more pronounced reduction in Alt a 1-induced IL-8 compared to blocking TLR2. We also examined if combining TLR2 and TLR4 blocking antibodies would have an additive effect in regards to dampening Alt a 1 induced IL-8 and found this to be the case. Pretreating cells with both TLR2 and TLR4 blocking antibodies completely abolished Alt a 1-induced IL-8 but was not statistically significant when compared to blocking TLR4 alone and may warrant further investigation. Collectively, these results suggested that Alt a 1-induced IL-8 in BEAS-2B cells is primarily dependent upon TLR4 with a minor contribution from TLR2.

### Engineered HEK 293 Cells Demonstrate Alt a 1-Induced NF-κB Signaling Is TLR 2 and 4 Dependent

**Figure 6:**
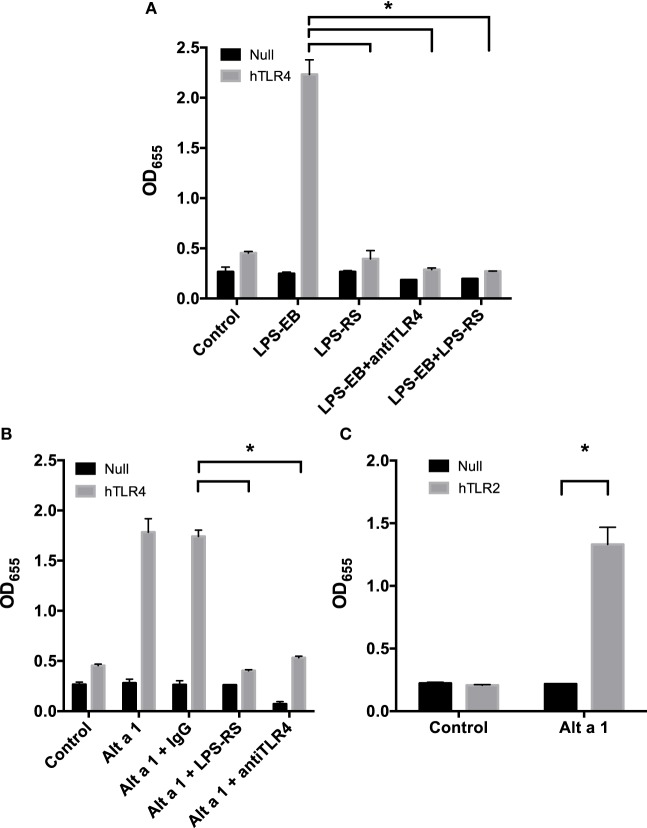
Alt a 1 activates toll-like receptor (TLR)2/4-dependent NF-κB signaling in engineered human embryonic kidney (HEK) cells. 2.5 × 10^4^ HEK-Blue hTLR4 **(A,B)**, HEK-Blue hTLR2 **(C)**, and HEK-Blue null cells **(A–C)** were plated in 96-well plates in 200 µL DMEM + 1% Pen/Strep/10% FBS. After 16 h at 37°C/5% CO_2_, the media was removed from the cells. Cells were then starved for 2 h in DMEM + 1% Pen/Strep. **(A)** Cells were pretreated with 5 ng/mL ultrapure LPS-RS, 10 µg/mL anti-hTLR4-IgG, or 10 µg/mL mouse IgG 1 control for an hour. Then cells were treated with 5 ng/mL ultrapure LPS-EB. Cells incubated with treatments in the incubator for 24 h. Afterward 20 µL of the cell supernatant was added to 180 µL QUANTI-Blue reagent. After incubation for 3 h at 37°C, the plate was read at 655 nM room temperature. Data are represented as mean (SD). After testing for homogeneity of variances, Tukey’s HSD was performed and adjusted (if applicable) (*p < 0.001). Comparisons shown are against LPS-EB (*p < 0.001). **(B)** Cells were pretreated with 5 ng/mL ultrapure LPS-RS, 10 µg/mL anti-hTLR4-IgG, or 10 µg/mL mouse IgG 1 control for an hour. Then cells were treated with 1 µg rAlt a 1. Cells incubated with treatments in the incubator for 24 h. Afterward, 20 µL of the cell supernatant was added to 180 µL QUANTI-Blue reagent. After incubation for 3 h at 37°C, the plate was read at 655 nM room temperature. Data are represented as mean (SD). After testing for homogeneity of variances, Tukey’s HSD was performed and adjusted (if applicable) (*p < 0.001). Comparisons shown are against Alt a 1 + IgG (*p < 0.001). **(C)** Cells were treated with 1 µg rAlt a 1. Data are represented as mean (SD). After testing for homogeneity of variances, Tukey’s HSD was performed and adjusted (if applicable) (*p < 0.001). Comparisons shown are against **(A)** LPS-EB, **(B)** Alt a 1 alone or Alt a 1 + IgG, and **(C)** Alt a 1 untreated null and hTLR2 cells and Alt a 1 treated null cells.

To further examine the importance of TLR4 in the initiation of the innate immune response to Alt a 1, we used HEK 293 cells engineered to express specific cell surface receptors such as TLR2, TLR4, or TLR5. These cells are also engineered with a reporter system whereby an NF-κB-dependent gene promoter is fused to a gene encoding a secreted form of alkaline phosphatase (SEAP). In initial experiments, HEK-Blue hTLR4 cells were used to determine if Alt a 1 could activate NF-κB signaling *via* TLR4 (Figure 6). We first optimized the systems using cells treated with a TLR4-specific agonist LPS (ultrapure TLR4-specific LPS-EB) and this resulted in high levels of SEAP (Figure 6A). This activity could be reduced when cells were pretreated with a TLR4 antagonist (LPS-RS which binds to TLR4 but does not induce a downstream signal), or with TLR4 blocking antibodies (Figure 6A).

Using this system, cells were pretreated with antibodies and then treated with rAlt a 1. Directly supporting our data from experiments with TLR4-specific siRNAs and blocking antibodies in BEAS-2B cells, the HEK-hTLR4 cells but not corresponding HEK-null control cells were responsive to rAlt a 1 (Figure 6B). Furthermore, this response could be almost completely abolished by directly blocking the TLR4 receptor using both TLR4 blocking antibodies and the TLR4 antagonistic ligand, LPS-RS. Non-blocking control antibodies had no effect on rAlt a 1-induced SEAP. We also performed similar experiments in hTLR2 HEK-blue cells. Results of these experiments indicated that Alt a 1 induced SEAP activity was dependent upon TLR2 (Figure 6C).

The specific mechanism for how Alt a 1 triggers both TLR2 and TLR4 is unclear. One explanation could lie in the requirement of co-receptor molecules for the receptors, such as CD14 and MD2. Experiments focusing upon co-receptor molecules for both TLRs were explored in HEK-hTLR2 and HEK-hTLR4 cells. CD14 associates with both TLR2 and TLR4 upon signaling. Another receptor, MD2, is more specific to TLR4. Therefore, we tested to see if these adaptors/co-receptors may be important for signaling using CD14 and MD2 blocking antibodies first in hTLR4 HEK-blue cells.

When CD14 was blocked in hTLR4 HEK-blue cells, signaling appeared to be reduced only slightly and was not statistically significant (Figure S1 in Supplementary Material). This suggested that Alt a 1 may trigger immunity in a CD14-independent manner to a large degree through TLR4. Results of these experiments also suggested that MD2 may not be important for the ability of Alt a 1 to signal through TLR4.

By contrast, blocking CD14 in HEK-hTLR2-blue cells treated with Alt a 1 appeared to cause a small but significant reduction in SEAP (Figure S2 in Supplementary Material). This indicated CD14 may be required for optimal signaling by Alt a 1 *via* TLR2. Blocking MD2 had no effect on HEK-hTLR2-blue cells treated with Alt a 1 indicating unlike CD14, MD2 is most likely not involved in Alt a 1 induced signaling *via* TLR2.

Finally, we investigated the MyD88-dependent TLR5 receptor using a similar approach. To ensure that the conditions of our system were compatible with HEK-hTLR5 cells, we first used bacterial flagellin from *S. typhimurium* (FLA-ST), a canonical ligand for TLR5 as a positive control. As expected, FLA-ST induced robust TLR5-dependent SEAP activity. In contrast to HEK-hTLR2 and HEK-hTLR4, HEK-hTLR5 cells treated with Alt a 1 showed no response indicating that Alt a 1 does not trigger a TLR5–MyD88-dependent response (data not shown).

## Discussion

Alt a 1 is the most relevant allergen from the fungus *A. alternata*. Over 90% of patients sensitized to *Alternaria* typically have specific IgE to Alt a 1, indicating that it is a major allergen (29, 30). Sequence and structural studies have indicated that Alt a 1 is a species-specific allergen with no known cross reactivity with other allergens. Its sequence and β-barrel structure is unique among fungal allergens (16–20). Insights into its function have been hypothesized. Because Alt a 1 localizes to the cell wall of spores, it has been predicted to play a role in the stability of the spore (31). *Alternaria* spores isolated from schools, libraries, and offices have been shown to be capable of secreting detectable levels of Alt a 1 (32). On plants, this protein may play a role in the pathogenesis of *Alternaria* spp. (33). This protein has the potential to bind quercetin/flavonol like molecules of plant and fungal origin (34). Alt a 1 has been shown to be localized in the cytoplasm and cell wall of spores and secreted extremely rapidly in media of physiologically relevant pH especially slightly acidic conditions (34). This suggests that following spore inhalation, Alt a 1 may be secreted into the airways quite rapidly. Based on these recent secretion studies it is possible that all individuals are chronically exposed to Alt a 1 even in the absence of fungal colonization of the airways. Studies examining the importance of Alt a 1 beyond the scope of its capability of being used in diagnostic procedures have not been conducted (21).

In this study, we report for the first time that Alt a 1 induces innate immune responses in bronchial epithelial cells *in vitro*. A combination of cell lines and primary cells (BEAS-2B, NHBE, DHBE) with complementary experimental approaches were used to determine that at least some aspects of Alt a 1 signaling are dependent upon PRRs and adaptor molecules including TLR2, TLR4, MYD88, and TIRAP. Moreover, this is the first study to show that a single, highly purified molecule from *Alternaria* induces innate immune responses.

Previously, most immunological studies surrounding *Alternaria* have employed potent extracts consisting of a complex mixture of proteins and speculatively other molecules from *Alternaria* which could include mycotoxins, other secondary metabolites, and cell wall fragments such as chitin, mannans, and β-1,3-glucans. The complexity and inconsistency of extract composition has made it challenging to define specific components contributing to the proinflammatory nature of these products. It is widely accepted that different culture and extraction conditions can lead to the variability in airway cell response to extracts (22, 35). In addition, extracts can have widely different allergen content (36). Expression of fungal allergens can even vary by strain (37).

It has been reported in several studies that protease activities in *Alternaria* extracts, especially serine, aspartic, and cysteine protease activity, are potent inducers of cytokines *in vitro* and *in vivo* (38–40). Using inhibitors, it was preciously shown that serine proteases activity from *Alternaria* induced TSLP and IL-33, potentially playing an important role in the development of allergic inflammation, airway disease, and severe asthma exacerbations (38). Aspartic protease activity was shown to activate eosinophils leading to degranulation (39). Aspartic protease activity was shown to induce the release of several other cytokines, including IL-6 and IL-8 (40). Despite the evidence for the role of *Alternaria* proteases found in extracts to be potent inducers of cytokines, no single purified protease has been identified and correlated with these activities. It is important to note that Alt a 1 has not been reported to possess any protease activity. Alt a 1’s enzymatic activity has been reported to be primarily esterase and phosphatase (14). While extracts have clearly defined a potential role that proteases play in the immune response of the airway to fungi, they have not allowed for the determination of the role that individual proteins of *Alternaria*, such as Alt a 1 may play in the context of allergic inflammation, sensitization, and development of more complex disorders such as asthma (41). It is interesting to speculate how Alt a 1 may contribute to the overall inflammatory response of *Alternaria* spores and hyphae in the context of other proinflammatory molecules found in *Alternaria* including proteases mentioned above, chitin, glucans, mannans, and other yet to be identified molecules. In this regard, we have created Alt a 1 knockout (KO) and overexpression mutants in *A. alternata* and have recently initiated *in vitro* and *in vivo* experiments comparing these mutants and wild-type (WT) spores for their ability to induce immune responses. Preliminary data from our lab indicate that mutant spores lacking Alt a 1 induce an overall lower innate immune response when compared to WT spores both *in vitro* (BEAS-2B cells) and in mouse *in vivo* models (Rumore et al., unpublished). Moreover, Alt a 1 overexpression mutants (secreting ~2.5 times as much Alt a 1 compared to WT) induce a dramatically higher innate immune response in these systems compared to Alt a 1 KO mutants or WT spores. Future experiments investigating the role of Alt a 1 in adaptive immunity *in vivo* are certainly warranted using these tools.

Although tremendous progress has been made over the past few decades regarding determining the mechanistic aspects of allergic inflammation, more research needs to be performed in innate immunity and its role in sensitization and exacerbation aspects of allergic diseases. Published studies have increasingly made it clear that TLRs are key players in innate immunity to a growing number of allergens. For example, the dust mite allergen, Der p 2, has been shown to mimic the activity of human and mouse MD2 in the presence of LPS to trigger a response through TLR4 (42) *in vitro* and *in vivo*. Der p 2 has been shown to induce TLR2–MYD88-dependent mediated innate immune signaling in nasal fibroblasts (43). In this study, we found the induction of several cytokines and chemokines by Alt a 1 in bronchial epithelial cells including GRO-a/b/g (CXCL1/2/3), IL-8, and MCP-1 (CCL2) and this was TLR2/4, MyD88, and TIRAP dependent. These cytokines and chemokines have been shown in many studies to play a role in monocyte, neutrophil, and fibroblast recruitment and angiogenesis in asthma and innate allergic inflammation (44–46). Studying if other cytokines and chemokines such as TSLP, IL-33, and IL-25 are induced by Alt a 1 and/or in different cell types may be the subject of future research. Our preliminary data strongly suggest that Alt a 1 is capable of inducing the release of IL-33 from NHBE cells and warrants further investigation in the future (data not shown).

More specifically, using gene knockdown approaches and complementary studies with blocking antibodies, we found that the ability of Alt a 1 to induce a potent cytokine response was dependent upon TLR2, TLR4, MyD88, and TIRAP. Treatment of engineered HEK-Blue Null, TLR2, TLR4, and TLR5 cells with Alt a 1 showed that TLR2 and TLR4-associated NF-κB signaling but not TLR5 is activated. Furthermore, incubation with TLR4 blocking antibodies and LPS-RS caused the abolishment of signaling in both HEK-Blue TLR4 and human bronchial epithelial airway (BEAS-2B) cells. In contrast to studies with Der p 2, the MD2 mimic that presents LPS to TLR4, our studies suggest that Alt a 1 does not function as an MD2 mimic because it is structurally unrelated to MD2 or Der p 2. Furthermore, we had virtually undetectable amounts of LPS in our protein preparation. LPS was a requirement for Der p 2 signaling *via* TLR4 (42).

In addition to MD2 and CD14, another important protein in TLR4 signaling is lipopolysaccharide-binding protein (LBP). LBP is mainly found in the serum and has been found to facilitate TLR4 signaling by carrying LPS to the receptor. In a study by Kato et al. comparing gene expression profiles in the presence and absence of serum, it was found that induction of cytokines like IL-8 and members of the GRO family in PBMCs was not dependent upon LBP being present, however, a subset of IFN-inducible genes was dependent upon LBP (47). The data from this study suggested that MyD88-dependent genes did not require LBP to be present but IRF-3 associated genes required LBP. In our experiments, we starved cells of serum prior to challenges with Alt a 1 and other ligands thus, most likely removing the vast majority of LBP present but still observed TLR4- and MyD88-dependent expression of cytokines like IL-8. If Alt a 1 functions as an LBP mimic, one would most likely not detect this in the context of our experimental design. As mentioned previously, we did not detect LPS in our protein preparations thus most likely ruling out the possibility that Alt a 1 could be an LPS carrier or binding protein mimicking MD2, CD14, or LBP. However, it is important to point out that even though our experiments using engineered HEK cells indicated that MD2 and CD14 are probably not major components of Alt a 1 induced signaling, overexpression of TLR2 and TLR4 receptors could mask the role of CD14 and MD2. Future experiments in BEAS-2B or other cell types are warranted to further elucidate the role of these adaptors in Alt a 1 induced immunity.

Another possibility to consider is contamination of our *Pichia*-produced protein preparations with ligands that may activate TLR2. However, there are no reports of *Pichia*-produced proteins harboring contaminants that activate TLR2. It has been shown that the allergen Der p 21 can induce IL-8 in BEAS-2B cells in a TLR2-dependent manner (48). However, rDer p 21 protein preparations used in this study were analyzed by MS and the authors did not report any contamination. The authors speculated that because Der p 21 is predicted to be a lipid binding protein it may carry an unknown ligand to TLR2. In the context of our study, it is possible that Alt a 1 directly binds to and activates TLR2 and TLR4 or liberates a ligand from the cell surface *via* its esterase and/or phosphatase enzymatic activity and will be the focus of future investigations.

Collectively, these findings provide new avenues for the study of allergic inflammation, the sensitization process, and the development of asthma and other allergic airway diseases especially to *Alternaria*.

## Author Contributions

TH and AR contributed equally to the manuscript (co-first authors), TH and AR designed and performed experiments, analyzed data, and contributed to writing of the paper, BH and XH designed and performed experiments and analyzed data, ML performed experiments and analyzed data, MC and SW created and provided experimental reagents critical for the study, LL and HK designed experiments and provided reagents, CL designed experiments, analyzed data, and contributed to writing of the paper.

## Conflict of Interest Statement

MC and SW were employed by the company Indoor Biotechnologies Inc., Charlottesville, VA. All other authors declare no competing interests. The handling Editor declared a shared affiliation, though no other collaboration, with one of the authors TH.

## References

[1] Gergen PJ Turkeltaub PC. The association of individual allergen reactivity with respiratory disease in a national sample: data from the second National Health and Nutrition Examination Survey, 1976-80 (NHANES II). J Allergy Clin Immunol (1992) 90:579–88. 10.1016/0091-6749(92)90130-T 1401641

[2] Halonen M Stern DA Wright AL Taussig LM Martinez FD. *Alternaria* as a major allergen for asthma in children raised in a desert environment. Am J Respir Crit Care Med (1997) 155:1356–61. 10.1164/ajrccm.155.4.9105079 9105079

[3] Salo PM Arbes SJ Jr Sever M Jaramillo R Cohn RD London SJ Exposure to *Alternaria alternata* in US homes is associated with asthma symptoms. J Allergy Clin Immunol (2006) 118:892–8. 10.1016/j.jaci.2006.07.037 17030243 PMC2080575

[4] O’Hollaren MT Yunginger JW Offord KP Somers MJ O’Connell EJ Ballard DJ Exposure to an aeroallergen as a possible precipitating factor in respiratory arrest in young patients with asthma. N Engl J Med (1991) 324:359–63. 10.1056/NEJM199102073240602 1987459

[5] Andersson M Downs S Mitakakis T Leuppi J Marks G. Natural exposure to *Alternaria* spores induces allergic rhinitis symptoms in sensitized children. Pediatr Allergy Immunol (2003) 14:100–5. 10.1034/j.1399-3038.2003.00031.x 12675755

[6] Peat JK Tovey E Mellis CM Leeder SR Woolcock AJ. Importance of house dust mite and *Alternaria* allergens in childhood asthma: an epidemiological study in two climatic regions of Australia. Clin Exp Allergy (1993) 23:812–20. 10.1111/j.1365-2222.1993.tb00258.x 10780887

[7] Peat JK Toelle BG Gray EJ Haby MM Belousova E Mellis CM Prevalence and severity of childhood asthma and allergic sensitisation in seven climatic regions of New South Wales. Med J Aust (1995) 163:22–6. 7609683 10.5694/j.1326-5377.1995.tb126083.x

[8] Hoffman DR Mould allergens. In: Al-Doory Y Domson JE editors. Mould Allergy. Philadelphia: Lea and Febiger (1984). p. 104–16.

[9] van Leeuwen WS Bronchial asthma in relation to climate. Proc R Soc Med (1924) 17:19–26. 10.1177/003591572401702507 PMC2201247 19984206

[10] Sanchez H Bush RK. A review of *Alternaria alternata* sensitivity. Rev Iberoam Micol (2001) 18:56–9. 15487907

[11] Breitenbach M Simon-Nobbe B The allergens of *Cladosporium herbarum* and *Alternaria alternata*. Chem Immunol (2002) 81:48–72. 10.1159/000058862 12102004

[12] Simon-Nobbe B Probst G Kajava AV Oberkofler H Susani M Crameri R IgE-binding epitopes of enolases, a class of highly conserved fungal allergens. J Allergy Clin Immunol (2000) 106:887–95. 10.1067/mai.2000.110799 11080711

[13] Weichel M Schmid-Grendelmeier P Flückiger S Breitenbach M Blaser K Crameri R. Nuclear transport factor 2 represents a novel cross-reactive fungal allergen. Allergy (2003) 58:198–206. 10.1034/j.1398-9995.2003.23822.x 12653793

[14] Barnes CS Pacheco F Landuyt J Rosenthal D Hu F Portnoy J. Production of a recombinant protein from *Alternaria* containing the reported N-terminal of the Alt a1 allergen. Adv Exp Med Biol (1996) 409:197–203. 10.1007/978-1-4615-5855-2_26 9095241

[15] Sáenz-de-Santamaría M Guisantes JA Martínez J. Enzymatic activities of *Alternaria alternata* allergenic extracts and its major allergen (Alt a 1). Mycoses (2006) 49:288–92. 10.1111/j.1439-0507.2006.01238.x 16784442

[16] Twaroch TE Curin M Sterflinger K Focke-Tejkl M Swoboda I Valenta R. Specific antibodies for the detection of *Alternaria* allergens and the identification of cross-reactive antigens in other fungi. Int Arch Allergy Immunol (2016) 170:269–78. 10.1159/000449415 27780168 PMC5321516

[17] Bowyer P Fraczek M Denning DW. Comparative genomics of fungal allergens and epitopes shows widespread distribution of closely related allergen and epitope orthologues. BMC Genomics (2006) 7:251. 10.1186/1471-2164-7-251 17029625 PMC1613252

[18] Crameri R Structural aspects of fungal allergens. Semin Immunopathol (2014) 37:117–21. 10.1007/s00281-014-0458-0 25413498

[19] Chruszcz M Chapman MD Osinski T Solberg R Demas M Porebski PJ *Alternaria alternata* allergen Alt a 1: a unique β-barrel protein dimer found exclusively in fungi. J Allergy Clin Immunol (2012) 130:241–7.e9. 10.1016/j.jaci.2012.03.047 22664167 PMC3391610

[20] Wagner GE Gutfreund S Fauland K Keller W Valenta R Zangger K Backbone resonance assignment of Alt a 1, a unique β-barrel protein and the major allergen of *Alternaria alternata*. Biomol NMR Assign (2014) 8:229–31. 10.1007/s12104-013-9489-z 23715812 PMC6597350

[21] Asturias JA Ibarrola I Ferrer A Andreu C López-Pascual E Quiralte J Diagnosis of *Alternaria alternata* sensitization with natural and recombinant Alt a 1 allergens. J Allergy Clin Immunol (2005) 115:1210–7. 10.1016/j.jaci.2005.02.012 15940136

[22] Paris S Fitting C Ramirez E Latgé JP David B. Comparison of different extraction methods of *Alternaria* allergens. J Allergy Clin Immunol (1990) 85:941–8. 10.1016/0091-6749(90)90081-E 2332567

[23] Aden E Weber B Bossert J Teppke M Frank E Wahl R Standardization of *Alternaria alternata*: extraction and quantification of alt a 1 by using an mAb-based 2-site binding assay. J Allergy Clin Immunol (1999) 104:128–35. 10.1016/S0091-6749(99)70124-7 10400850

[24] Implementing a Class of Permutation Tests. The coin Package | Hothorn. J Stat Softw (2017). Available from: https://www.jstatsoft.org/article/view/v028i08 (accessed January 8, 2017).

[25] An R Companion to Applied Regression | SAGE Publications Inc (2017). Available from: https://us.sagepub.com/en-us/nam/an-r-companion-to-applied-regression/book233899 (accessed January 8, 2017).

[26] Ritz C Baty F Streibig JC Gerhard D. Dose-response analysis using R. PLoS One (2015) 10:e0146021. 10.1371/journal.pone.0146021 26717316 PMC4696819

[27] Hothorn T Bretz F Westfall P. Simultaneous inference in general parametric models. Biom J (2008) 50:346–63. 10.1002/bimj.200810425 18481363

[28] Object-oriented computation of sandwich estimators. J Stat Softw (2017). Available from: https://www.jstatsoft.org/article/view/v016i09 (accessed January 8, 2017).

[29] Yunginger JW Jones RT Nesheim ME Geller M Studies on *Alternaria* allergens. J Allergy Clin Immunol (1980) 66:138–47. 10.1016/0091-6749(80)90061-5 7190582

[30] Kleine-Tebbe J Worm M Jeep S Matthiesen F Løwenstein H Kunkel G. Predominance of the major allergen (Alt a I) in *Alternaria* sensitized patients. Clin Exp Allergy (1993) 23:211–8. 10.1111/j.1365-2222.1993.tb00884.x 7682472

[31] Twaroch TE Predominant localization of the major *Alternaria* allergen Alt a 1 in the cell wall of airborne spores. J Allergy Clin Immunol (2012) 129:1148–9. 10.1016/j.jaci.2011.10.008 22078468

[32] Skóra J Otlewska A Gutarowska B Leszczyńska J Majak I Stępień Ł. Production of the allergenic protein Alt a 1 by *Alternaria* isolates from working environments. Int J Environ Res Public Health (2015) 12:2164–83. 10.3390/ijerph120202164 25689994 PMC4344718

[33] Cramer RA Lawrence CB. Cloning of a gene encoding an Alt a 1 isoallergen differentially expressed by the necrotrophic fungus *Alternaria brassicicola* during *Arabidopsis* infection. Appl Environ Microbiol (2003) 69:2361–4. 10.1128/AEM.69.4.2361-2364.2003 12676721 PMC154767

[34] Garrido-Arandia M Silva-Navas J Ramírez-Castillejo C Cubells-Baeza N Gómez-Casado C Barber D Characterisation of a flavonoid ligand of the fungal protein Alt a 1. Sci Rep (2016) 6:33468. 10.1038/srep33468 27633190 PMC5025882

[35] Kim JH Harvey LA Evans AL Byfield GE Betancourt DA Dean TR. Biological responses of Raw 264.7 macrophage exposed to two strains of *Stachybotrys chartarum* spores grown on four different wallboard types. Inhal Toxicol (2016) 28:303–12. 10.3109/08958378.2016.1170909 27097835

[36] De Vouge MW Thaker AJ Zhang L Muradia G Rode H Vijay HM. Molecular cloning of IgE-binding fragments of *Alternaria alternata* allergens. Int Arch Allergy Immunol (1998) 116:261–8. 10.1159/000023954 9693275

[37] Martínez J Gutiérrez A Postigo I Cardona G Guisantes J. Variability of Alt a 1 expression by different strains of *Alternaria alternata*. J Investig Allergol Clin Immunol (2006) 16:279–82. 17039665

[38] Snelgrove RJ Gregory LG Peiró T Akthar S Campbell GA Walker SA *Alternaria*-derived serine protease activity drives IL-33–mediated asthma exacerbations. J Allergy Clin Immunol (2014) 134:583–92.e6. 10.1016/j.jaci.2014.02.002 24636086 PMC4152000

[39] Matsuwaki Y Wada K White TA Benson LM Charlesworth MC Checkel JL Recognition of fungal protease activities induces cellular activation and eosinophil-derived neurotoxin release in human eosinophils. J Immunol (2009) 183:6708–16. 10.4049/jimmunol.0901220 19864598 PMC2843542

[40] Matsuwaki Y Wada K White T Moriyama H Kita H. *Alternaria* fungus induces the production of GM-CSF, interleukin-6 and interleukin-8 and calcium signaling in human airway epithelium through protease-activated receptor 2. Int Arch Allergy Immunol (2012) 158:19–29. 10.1159/000337756 22627362 PMC3395436

[41] Kustrzeba-Wójcicka I Siwak E Terlecki G Wolańczyk-Mędrala A Mędrala W. *Alternaria alternata* and its allergens: a comprehensive review. Clin Rev Allergy Immunol (2014) 47:354–65. 10.1007/s12016-014-8447-6 25205364

[42] Trompette A Divanovic S Visintin A Blanchard C Hegde RS Madan R Allergenicity resulting from functional mimicry of a toll-like receptor complex protein. Nature (2009) 457:585–8. 10.1038/nature07548 19060881 PMC2843411

[43] Chiou Y-L Lin C-Y. Der p2 activates airway smooth muscle cells in a TLR2/MyD88-dependent manner to induce an inflammatory response. J Cell Physiol (2009) 220:311–8. 10.1002/jcp.21764 19326394

[44] Keglowich L Roth M Philippova M Resink T Tjin G Oliver B Bronchial smooth muscle cells of asthmatics promote angiogenesis through elevated secretion of CXC-chemokines (ENA-78, GRO-α, and IL-8). PLoS One (2013) 8:e81494. 10.1371/journal.pone.0081494 24339939 PMC3855263

[45] Singh SR Sutcliffe A Kaur D Gupta S Desai D Saunders R CCL2 release by airway smooth muscle is increased in asthma and promotes fibrocyte migration. Allergy (2014) 69:1189–97. 10.1111/all.12444 24931417 PMC4215601

[46] Roy RM Wüthrich M Klein BS. Chitin elicits CCL2 from airway epithelial cells and induces CCR2-dependent innate allergic inflammation in the lung. J Immunol (2012) 189:2545–52. 10.4049/jimmunol.1200689 22851704 PMC3424300

[47] Kato A Ogasawara T Homma T Saito H Matsumoto K. Lipopolysaccharide-binding protein critically regulates lipopolysaccharide-induced IFN-beta signaling pathway in human monocytes. J Immunol (2004) 172(10):6185–94. 10.4049/jimmunol.172.10.6185 15128806

[48] Pulsawat P Theeraapisakkun M Nony E Le Mignon M Jain K Buaklin A Characterization of the house dust mite allergen Der p 21 produced in *Pichia pastoris*. Protein Expr Purif (2014) 101:8–13. 10.1016/j.pep.2014.05.001 24874917

