# Supplementary figures and images for: Innate Immunity Induced by the Major Allergen Alt a 1 From the Fungus Alternaria Is Dependent Upon Toll-Like Receptors 2/4 in Human Lung Epithelial Cells
Source: Front Immunol. 2018 Jul 30;9:1507. doi: 10.3389/fimmu.2018.01507 (PMC6072870; doi:10.3389/fimmu.2018.01507)

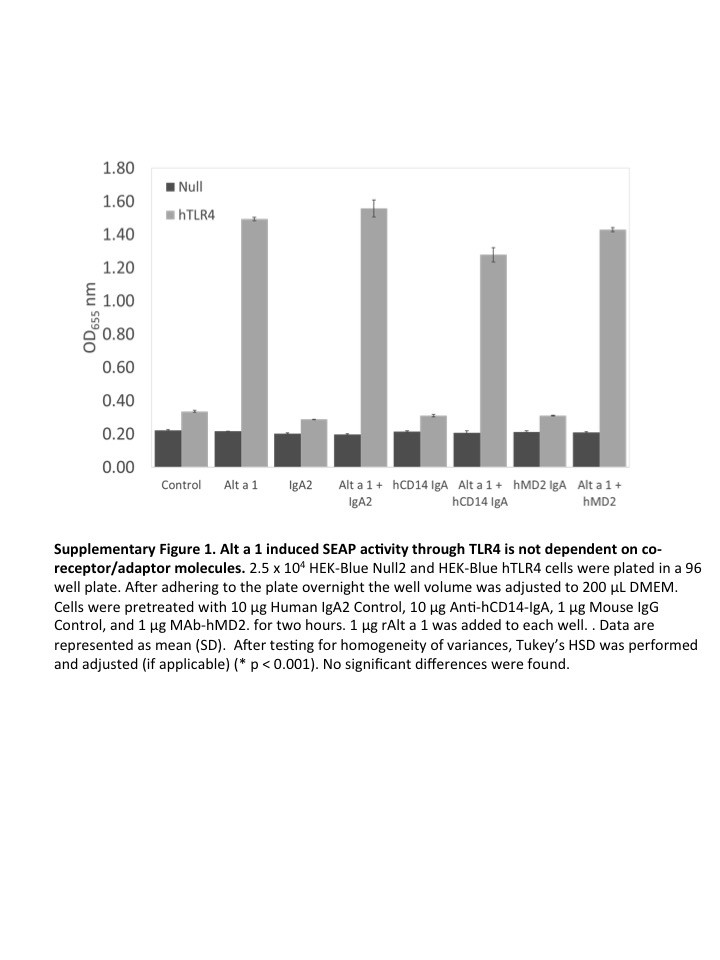

Supplement: Supplementary file 1 [file image_1.jpeg]

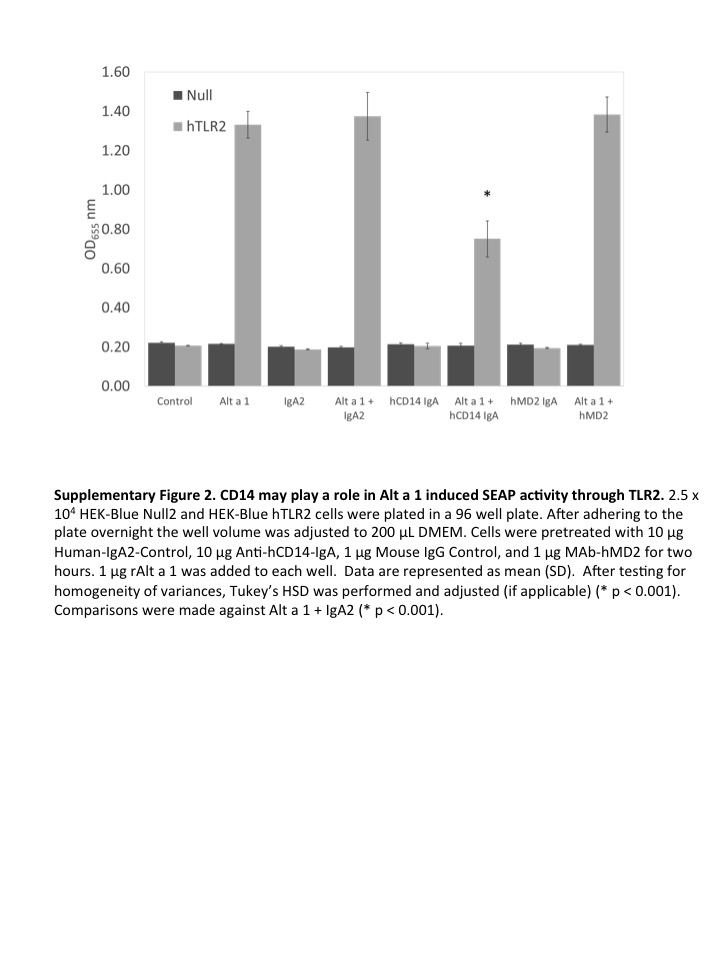

Supplement: Supplementary file 2 [file image_2.jpeg]
